# Is cognitive lifestyle associated with depressive thoughts and self-reported depressive symptoms in later life?

**DOI:** 10.1007/s10433-015-0359-7

**Published:** 2015-12-15

**Authors:** Carol Opdebeeck, Catherine Quinn, Sharon M. Nelis, Linda Clare

**Affiliations:** 1 Research in Ageing and Cognitive Health (REACH), School of Psychology, Bangor University, Bangor, Gwynedd LL572AS UK; 2 REACH: The Centre for Research in Ageing and Cognitive Health, School of Psychology, University of Exeter, Exeter, EX44QG UK

**Keywords:** Education, Occupation, Cognitive leisure activities, Cognitive reserve

## Abstract

Key components of cognitive lifestyle are educational attainment, occupational complexity and engagement in cognitively stimulating leisure activities. Each of these factors is associated with experiencing fewer depressive symptoms in later life, but no study to date has examined the relationship between overall cognitive lifestyle and depressive symptoms. This task is made more complex because relatively few older participants in cross-sectional studies will be currently experiencing depression. However, many more will show evidence of a depressive thinking style that predisposes them towards depression. This study aimed to investigate the extent to which cognitive lifestyle and its individual components are associated with depressive thoughts and symptoms. Two hundred and six community-dwelling participants aged 65+ completed the depressive cognitions scale, the geriatric depression scale and the lifetime of experiences questionnaire, which assesses cognitive lifestyle. Correlational analysis indicated that each of the individual lifestyle factors—education, occupational complexity and activities in young adulthood, mid-life and later life—and the combined cognitive lifestyle score was positively associated with each other and negatively with depressive symptoms, while all except education were negatively associated with depressive thoughts. Depressive thoughts and symptoms were strongly correlated. Cognitive lifestyle score explained 4.6 % of the variance in depressive thoughts and 10.2 % of the variance in depressive symptoms. The association of greater participation in cognitive activities, especially in later life, with fewer depressive symptoms and thoughts suggests that preventive interventions aimed at increasing participation in cognitively stimulating leisure activity could be beneficial in decreasing the risk of experiencing depressive thoughts and symptoms in later life.

## Introduction

Depressive symptoms in later life are associated with a number of negative outcomes; these can include increased disability, frailty and loss of independence in older age (Agüero-Torres et al. 2002; Covinsky et al. 2010; Lenze et al. 2001; Mezuk et al. 2012; Reynolds et al. 2008). In addition, the experience of depression in later life is associated with poorer cognitive function and increased risk of developing cognitive impairment and dementia (Reppermund et al. 2011; Dotson et al. 2010; Yates et al. 2013). However, the association between depression and cognitive impairment and dementia is complex and it currently remains unclear whether depression is a risk factor for dementia or a prodromal symptom, or whether there is some underlying mechanism that is shared between depression and dementia (Byers and Yaffe 2011; Korczyn and Halperin 2009; Leonard 2007). Given the negative outcomes associated with depression and its complex association with late-life cognitive ability, it is of interest to investigate potentially modifiable factors that are associated with increased risk of both depression and cognitive impairment or dementia in later life.

Analysis of data from several European cities has indicated a 12.3 % prevalence of depression for community-dwelling people aged above 65 (Copeland et al. 2004). While the prevalence of depression in later life is high, cross-sectional research generally only captures those who are currently experiencing depressive symptoms, and not those who have experienced depressive symptoms in the past or who may experience them in the future (Crawford et al. 2001). However, thought processes related to lowered mood, such as negative thoughts or depressive cognitions focusing on self, the world and the future, are thought to increase the risk of experiencing a depressive episode in both younger and older people, and these are more persistent trait variables (Beck 2002; Evans et al. 2005; Zauszniewski 1997; Zauszniewski and Rong 1999). Depressive symptoms refer to those symptoms observed in clinically diagnosed depression, and this term is frequently used when symptoms are assessed via a screening tool which cannot provide a clinical diagnosis of depression. The experience of mild depressive symptoms and the depressive thought patterns associated with depression are likely to be more common in community-dwelling older people than clinically diagnosed depression (Beekman et al. 1999). These depressive symptoms and thought patterns, like clinical depression, are associated with cognitive impairment and functional disabilities (Zauszniewski and Rong 1999; Vinkers et al. 2004; Wilson et al. 2003; Yen et al. 2011). Therefore, it is important to consider potentially modifiable factors associated not only with clinically diagnosed depression but also with mild depressive symptoms and those depressive thoughts that may in turn increase the risk of experiencing an episode of clinical depression. One such factor may be cognitive lifestyle.

An active cognitive lifestyle is related to better cognition in later life and a reduced risk of cognitive impairment and dementia, and is thought to increase cognitive reserve (Foubert-Samier et al. 2012; Opdebeeck et al. 2015; Stern 2009; Tucker and Stern 2011; Valenzuela and Sachdev 2006a, b). The key components of an active cognitive lifestyle are educational level, a complex or challenging occupation, usually indicated through higher status or professional occupations and engagement in cognitively stimulating leisure activities (Stern 2009). Cognitive reserve is the concept that was first proposed to account for observed individual differences in levels of neuropathology and corresponding cognitive functioning, and is thought to be the consequence of engagement in cognitively stimulating activities across the lifespan (for reviews see Nucci et al. 2011; Richards and Deary 2005; Richards and Sacker 2003; Sánchez Rodríguez et al. 2011; Stern 2009; Tucker and Stern 2011; Whalley et al. 2006). As several researchers have proposed that there may be a shared underlying mechanism for both dementia and depression in later life (Byers and Yaffe 2011; Korczyn and Halperin 2009; Leonard 2007), cognitive reserve may also help to protect against the experience of depressive symptoms and depressive thoughts. In Paulson et al. (2014), a higher level of education was protective against the negative effects of cerebrovascular burden on levels of depressive symptoms at baseline. A recent review of the neurobiology of later life depression argued the case for cognitive reserve as a protective factor against late-life depression but suggested that further research is required (Weisenbach and Kumar 2014).

There is consensus from previous research that higher educational level and greater participation in leisure activities are associated with fewer depressive symptoms in later life (Adams et al. 2011; Bjelland et al. 2008; Glass et al. 2006; Hong et al. 2009; Jenkins 2011; Ladin 2008; Lorant et al. 2003; Murrell et al. 2003; Narushima et al. 2013; Ross and Mirowsky 2006, 2010). Fewer studies have assessed the relationship between holding a higher status or more cognitively complex occupation and depressive symptoms in later life, and to date findings are mixed, perhaps partly due to methodological differences. Alvarado and colleagues (2007) reported that primary lifetime occupation was associated with late-life depression in two out of six cities in Latin America and the Caribbean, with less complex work associated with a greater risk of depression. However, Lindesay et al. (1989) found no association between the last occupation participants held and the presence of depression in later life. The associations of education, cognitively stimulating leisure activity and occupation with depressive thoughts, which are likely to increase the risk of experiencing a depressive episode, have not been investigated previously. Additionally, no study to date has combined these key components of an active cognitive lifestyle to assess the associations between experiences at different life stages and depressive symptoms in older people.

Current evidence suggests that education is the strongest protective factor against cognitive decline and dementia, although engagement in a cognitively challenging occupation, and in cognitively stimulating leisure activities, is also associated with better cognitive function and a reduced risk of cognitive decline and dementia (Opdebeeck et al. 2015; Valenzuela and Sachdev 2006a, b). The current evidence indicates that education and cognitively stimulating leisure activities are more consistently associated with levels of depressive symptoms than occupation. However, the association between occupational level and depressive symptoms in older people has not been investigated to the same extent. No previous study has assessed the associations between any of these indicators of an active cognitive lifestyle across the lifespan and depressive thoughts. It is important to consider a lifespan perspective as it is probable that, rather than any one of these three components of an active cognitive lifestyle acting in isolation, they interact and accumulate across the lifespan to affect mood and cognition in later life. However, there is no evidence to date as to whether lifetime cognitive activity or cognitive activity at any one life stage is of greater importance in explaining variance in depressive thoughts or symptoms in older people. Additionally, it is unclear whether proximal or distal factors are more relevant.

The paucity of current evidence regarding the association of educational level, occupational complexity and engagement in cognitive activity with depressive thoughts makes it difficult to predict the strength of any associations. As depressive thoughts are highly associated with depression, which is known to be negatively associated with these factors, a negative association can be hypothesised. However, it is probable that these negative thoughts are more pervasive and consistent than depressive symptoms or episodes and therefore it is possible that cognitive lifestyle components exert a smaller influence on depressive thoughts. It should also be noted that a number of other factors not considered here may influence the level of depressive symptoms in later life, most notably adverse life events, illness, or bereavement (Bruce 2002; Cole and Dendukuri 2003). Nevertheless, the purpose of the current study was to assess the extent to which an active cognitive lifestyle across the lifespan, which has previously been associated with better cognitive function and a reduced risk of dementia, is associated with depressive thoughts and symptoms in older people. The study had two specific aims:

1. To assess whether a validated measure of participation in cognitively stimulating activities across the lifespan, yielding a cognitive lifestyle score that is frequently considered a proxy measure of cognitive reserve, accounts for a significant amount of variance in depressive thoughts and symptoms in community-dwelling older people.
2. To investigate whether there was a cumulative effect, through direct or indirect pathways, of the key individual components of an active cognitive lifestyle, namely education, occupational complexity and participation in cognitively stimulating leisure activities at different times across the lifespan, in accounting for variance in depressive thoughts and symptoms in later life.

## Method

### Design

This study was a cross-sectional observational study involving self-report questionnaires and a brief cognitive assessment. Written informed consent was obtained from each participant. Ethical approval for the research was granted by the School of Psychology Ethics and Research Committee at Bangor University.

### Participants

The inclusion criteria required that participants should be above 65 years of age and in good health according to self-report, with no self-reported history of neurological disorder, psychosis or cognitive impairment. Participants were recruited using a purposive snowball sampling method from Agewell centres, active retirement groups, over 50 s clubs and church groups and through responses to flyers advertising the study, in the UK and Republic of Ireland. An a priori power analysis (Cohen 1992) indicated that a minimum sample size of 107 would provide sufficient power to detect a medium effect at *α* = .05. Two hundred and seven healthy, community-dwelling older people were recruited. Trained researchers met with participants at either their own home or the university for a single-testing session during which the self-completion questionnaires were completed, and the Lifetime of Experiences Questionnaire and neuropsychological assessment were administered. One participant chose to withdraw at the time of testing and is not included in the analyses.

### Measures

Demographic and background details recorded were age, gender and self-reported current illnesses (see Table 1).

**Table 1:** Demographic information (*n* = 206)

	*n*/Mean	Range
Gender		
Male	68 (33 %)	
Female	138 (67 %)	
Age	72.79 (6.46)	65–93
Education (years)	14.31 (3.91)	2–27.5
Marital status		
Married	122 (59.2 %)	
Never married/divorced	26 (12.6 %)	
Widowed	58 (28.2 %)	
No. of current illnesses	1.08 (1.03)	0–5
Most common medical conditions reported		
Arthritis/osteoporosis	46 (22.3 %)	
High/low blood pressure	38 (18.4 %)	
Diabetes	19 (9.2 %)	
Heart problem	14 (6.8 %)	
Stomach problem	11 (5.3 %)	
Thyroid problem	10 (4.9 %)	
High cholesterol	7 (3.4 %)	

Depressive thoughts were assessed using the depressive cognitions scale (DCS; Zauszniewski 1995), an 8-item self-rating questionnaire designed to measure thinking styles associated with depression. Each item reflects one of the key themes occurring in depressive cognitions (helplessness, hopelessness, purposelessness, worthlessness, powerlessness, loneliness, emptiness and meaninglessness). Scores can range from 0 to 40, with higher scores indicating a higher level of depressive cognitions. Scores of 0–6 indicate normal levels of depressive cognitions, while scores of 7–40 indicate serious depressive cognitions and a greater risk of depression (Zauszniewski and Bekhet 2012). The test is valid and reliable, with Cronbach’s alpha ranging from .75 to .88 across diverse populations (Zauszniewski and Bekhet 2011) and of .88 in the current study.

Levels of depression were assessed using the Geriatric Depression Scale (15 items short form; GDS-15; Yesavage and Sheikh 1986). This measure is a screening instrument that assesses depressive symptoms in older people through 15 self-report questions with yes/no responses. A lower score suggests fewer depressive symptoms. Scores of 0–4 indicate no evidence of depression, scores of 5–9 indicate possible mild depression and scores of 10–15 indicate possible moderate to severe depression (Alden et al. 1989). The GDS-15 has good internal consistency, with a Cronbach’s alpha of .83 in previous research (Chiang et al. 2009) and .72 in the current study. It has good test–retest reliability and is valid against other clinical measures of depression (Yesavage and Sheikh 1986).

The lifetime of experiences questionnaire (LEQ; Valenzuela and Sachdev 2007) yields a cognitive lifestyle score that reflects engagement in complex cognitive activity across the lifespan. It was developed as a means of quantifying the key life experiences thought to contribute to cognitive reserve, specifically education, occupational complexity and engagement in leisure activities. The LEQ covers three life stages (young adulthood, mid-life and later life). The section for each life stage contains both age-specific and general questions. The scores for the specific questions relating to each life stage are weighted to allow for an equal contribution of experiences from across the lifespan. The general questions are the same for all life stages and assess the average frequency with which participants engaged in seven activities: visiting family/friends; developing/practicing an artistic pastime such as writing; playing a musical instrument; engaging in physical activity; reading; speaking a foreign language and travel. The scores for these questions are added to the scores for the age-specific questions. Higher scores indicate a more active cognitive lifestyle, considered to be associated with higher cognitive reserve. The young adulthood score comprises scores regarding the level of education attained prior to the age of 30 and scores for the general questions.

The mid-life score comprises scores for occupational complexity and education undertaken between the ages of 30 and 65 (or retirement) and scores for the general questions. Occupation type is scored in five-year increments from the age of 30 to 65; in this study occupation was rated using Office for National Statistics (2010) classifications. The final occupational complexity score also comprises scores for managerial experience, which is thought to increase the complexity or challenge of the occupation. The later life section comprises scores for the general questions and for frequency of engagement in activities specific to this time of life (e.g. frequency of charity/volunteer work, membership of social clubs or groups, methods of seeking information about the world, number of different types of material read). Additional scores are given for any formal education or paid work undertaken in later life. Reports on the reliability of the measure suggest that it is variable, with Cronbach’s alpha ranging from .43 to .84 (Valenzuela and Sachdev 2007) and .56 to .81 in the current study. This is to be expected given that the measure assesses a number of life experiences that are not necessarily theoretically or conceptually related; indeed, the LEQ is designed to assess a wide variety of unrelated activities to capture the types of activity undertaken by different people. The LEQ has high construct validity, concurrent validity and clinical validity as well as good test–retest reliability (Valenzuela and Sachdev 2007).

The Addenbrooke’s Cognitive Examination III (ACE-III; Hsieh et al. 2013) was used to characterise the sample in terms of cognitive function. The ACE-III is a cognitive screening tool that assesses five cognitive domains: attention and orientation, memory, verbal fluency, language and visuospatial skills. The ACE-III is highly correlated with its previous version, the ACE-R, which incorporated the Mini Mental State Examination (Folstein et al. 1975) and was shown to have high specificity and sensitivity (Hsieh et al. 2013). The maximum total score is 100, with higher scores indicating better performance. In the current study, the ACE-III had good reliability with a Cronbach’s alpha of .78.

### Data analysis

Data were analysed using SPSS v.20. Pearson’s r correlations were calculated to investigate associations between the variables. Simple regression analyses were used to address the first aim of this study and indicate whether cognitive reserve, as indicated by a validated measure of participation in complex mental activities across the lifespan, accounted for a significant amount of variance in depressive thoughts and symptoms. Details of participants’ years of education, cognitively stimulating activities undertaken in young adulthood and mid-life, the total occupational complexity scores from age 30 to 65 and later life activity scores were taken from the LEQ to allow for an assessment of the cumulative effects of life experiences. Hierarchical multiple regressions were then conducted to assess whether there was a cumulative effect of activity engagement across the lifespan on the amount of variance in depressive thoughts and depressive symptoms that were accounted for. Taking depressive thoughts and depressive symptoms separately, in each case variables reflecting activity engagement at different stages of the lifespan were entered sequentially into the regression model. Factors reflecting engagement in complex mental activity in young adulthood—years of education and activities—were entered first, occupational complexity scores and activities undertaken in mid-life were added in the second step, and a score for participation in activities in later life was added in the third and final step. Finally, a parsimonious path analysis was conducted using AMOS v.22, with only the significant pathways between variables included, to demonstrate the additive effect of experiences across the lifespan on depressive thoughts and symptoms. Chi squared, the comparative fit index (CFI) and the root mean square error of approximation (RMSEA) were taken as indices of the fit of the model to the data.

All regression analyses are reported using adjusted *R* ^*2*^. Collinearity statistics were examined to ensure there were no issues of multicollinearity in the hierarchical regressions. There were no significant differences between males and females in either levels of depressive cognitions or self-reported experience of depressive symptoms, and therefore gender was not added as a covariate in the analysis. Similarly, age or cognitive function did not add significantly to any of the regression models and resulted in very minor changes in standardised beta values, and these are therefore not reported in these analyses.

## Results

Participants were 206 community-dwelling older people. Demographic details and the most commonly reported medical conditions are shown in Table 1. Scores for all questionnaire measures are shown in Table 2. 38 % (*n* = 78) of participants reported clinical levels of depressive thoughts, while 8 % (*n* = 17) reported mild levels of depressive symptoms and 1 % (*n* = 2) reported moderate to severe levels of depressive symptoms. As participants could not be divided into equal-sized groupings by levels of depressive thoughts or symptoms, both depressive thoughts and symptoms were considered as continuous variables, with higher scores on the DCS indicating higher experience of depressive thoughts and higher scores on the GDS indicating greater experience of the symptoms associated with depression.

**Table 2:** Means, standard deviations and ranges for all measures (*n* = 206)

	Possible range	Mean (SD)	Min–Max
Depressive cognitions (DCS)	0–40	5.80 (5.16)	0–34
Depressive symptoms (GDS)	0–15	1.63 (2.01)	0–12
LEQ (CR) Total	0–∞	101.99 (22.43)	44.20–159.60
LEQ Young adulthood activities	0–35	20.20 (4.62)	7–31
LEQ occupation	0–∞	55.25 (22.28)	13–95
LEQ Mid-life activities	0–35	20.62 (4.12)	8–30
LEQ Late-life activities	0–∞	45.54 (8.04)	26–65
ACE-III	0–100	90.66 (6.62)	63–100

*DCS* depressive cognitions scale, *GDS* geriatric depression scale, *LEQ (CR)* lifetime of experiences questionnaire (cognitive reserve), *ACE-III* Addenbrooke’s cognitive examination III; for the LEQ and ACE-III, a higher score indicates a better score. For depressive symptoms and depressive cognitions, a higher score indicates greater symptoms

∞, no max score available

Pearson’s r correlations between variables are summarised in Table 3. There were significant small to moderate negative correlations between depressive thoughts and the total cognitive lifestyle score (LEQ), young adulthood activities, occupational complexity scores, mid-life activities and later life activities. Depressive symptoms had significant moderate negative correlations with the total cognitive lifestyle score (LEQ) and later life activities, and small, but significant, negative correlations with all the remaining individual elements of the cognitive lifestyle score and cognitive function. Additionally, there were significant small to moderate positive correlations between all the cognitive lifestyle variables and cognitive function.

**Table 3:** Correlations between depressive symptoms, depressive cognitions, variables representing engagement in cognitive activity across the lifespan, and cognitive function

	DCS	GDS	LEQ (CR)	Education	Young adulthood activities	Occupation	Mid-life activities	Later life activities	Cognition (ACE-III)
DCS		.618**	−.225**	−.035	−.226**	−.183**	−.220**	−.338**	.030
GDS			−.326**	−.168*	−.183**	−.250**	−.256**	−.393**	−.221**
LEQ (CR)				.695**	.687**	.683**	.682**	.715**	.452**
Education					.315**	.453**	.213**	.312**	.480**
Young adult activities						.266**	.705**	.529**	.152*
Occupation							.280**	.366**	.253**
Mid-life activities								.656**	.183**
Later life activities									.300**
Cognition (ACE-III)									

*DCS* depressive cognitions scale, *GDS* geriatric depression scale, *LEQ (CR)* lifetime of experiences questionnaire (cognitive reserve), *ACE-III* Addenbrooke’s Cognitive Examination III

* Indicates significant at *p* < .05, ** Indicates significant at *p* < .01

As the independent variables were moderately to strongly correlated, collinearity statistics were examined. Tolerance and the variance inflation factors (VIF) were all within accepted limits indicating that there were no issues of multicollinearity in the following regression analyses (Robinson and Schumacker 2009).

In relation to the first aim of this study, the total cognitive lifestyle score from the LEQ accounted for 4.6 % of the variance in depressive thoughts (*F* = 10.85, *p* = .001) with a standardised beta coefficient of −.225, suggesting that higher cognitive lifestyle score was associated with lower levels of depressive thoughts. The total cognitive lifestyle score accounted for 10.2 % of the variance in self-reported experience of depressive symptoms (*F* = 24.32, *p* < .001) with a standardised beta coefficient of −.326, suggesting that higher cognitive lifestyle score was associated with experiencing fewer depressive symptoms.

In relation to the second aim of the study, the hierarchical regressions indicated that as scores for elements of the cognitive lifestyle score at each life stage were added to the model, a greater amount of variance was explained; however, not all predictors were independently significant (Table 4).

**Table 4:** Hierarchical regression analyses for depressive thoughts and depressive symptoms

	Depressive thoughts					Depressive symptoms				
	*β*	*t*	∆*R* ^2^	*R* ^2^ change	*F* change	*β*	*t*	∆*R* ^2^	*R* ^2^ change	*F* change
Step 1			.043**	.052	5.59**			.038**	.047	5.00**
Education	.039	0.55				−.123	−1.70			
Young adult activities	−.228***	−3.30				−.144*	−1.99			
Step 2			.061**	.027	2.91			.085***	.056	6.30**
Education	.102	1.30				−.058	−0.75			
Young adult activities	−.155	−1.57				.038	−0.39			
Occupation	−.161*	−2.06				−.172*	−2.27			
Mid-life activities	−.084	−0.86				−.223*	−2.34			
Step 3			.113***	.056	12.80***			.150***	.068	16.32***
Education	.135	1.77				−.020	−0.28			
Young adult activities	−.117	−1.21				.079	0.84			
Occupation	−.111	−1.44				−.118	−1.59			
Mid-life activities	.066	0.64				−.053	−0.53			
Later life activities	−.318***	−3.58				−.352***	−4.04			

* Indicates *p* < .05 ** Indicates *p* < .01 *** Indicates *p* < .001

With regard to depressive thoughts, in the first step, education and young adulthood activities together explained 4.3 % of the variance, with only activities an independently significant predictor of depressive thoughts. The addition of mid-life experiences did not result in a significant *F* change, but did increase the variance explained to 6.1 %; only occupational complexity was an independently significant predictor of depressive thoughts. The full model accounted for 11.3 % of the variance in depressive thoughts (*F* = 6.20, *p* < .001). However, it is probable that most of this variance was accounted for by later life activities, the only independently significant predictor of depressive thoughts in the full model.

With regard to self-reported experience of depressive symptoms, addition of the elements of the cognitive lifestyle score for each life stage significantly increased the amount of variance explained by the model. As was the case for depressive thoughts, only young adulthood activities were an independently significant predictor of depressive symptoms in the first step, with 3.8 % of variance explained. In the second step, there was an increase to 8.5 % in the variance accounted for, with both occupational complexity scores and mid-life activities emerging as independently significant predictors of depressive symptoms. The full model accounted for 15 % of the variance in depressive symptoms (*F* = 8.22, *p* < .001). However, as with depressive thoughts, only later life activities were an independently significant predictor of depressive symptoms in the full model. This indicates that greater engagement in activities in later life is associated with less depressive thoughts and symptoms.

**Fig. 1:**
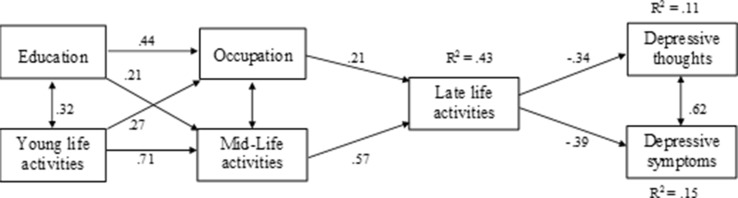
Path analysis model illustrating the significant pathways between individual components of cognitive lifestyle to depressive thoughts and symptoms

Figure 1 demonstrates the path analysis model that illustrates the inter-relationships between components of an active cognitive lifestyle and the direct and indirect associations with depressive thoughts and symptoms. The model also demonstrates the strong association between depressive thoughts and depressive symptoms. The model indicates that education and activities in early life interact and contribute to occupation and activities in mid-life that in turn interact and contribute to later life activities that have a direct negative effect on depressive thoughts and symptoms. The models for both depressive thoughts and symptoms show good fit to the data (depressive thoughts: *χ* ^*2*^ = 11.76, df = 6, *p* = .068; CFI = .983; RMSEA = .068; depressive symptoms, *χ* ^*2*^ = 10.90, df = 6, *p* = .092; CFI = .986; RMSEA = .063, *p* = .089).

## Discussion

The first aim of the current study was to assess whether a validated measure of participation in cognitively stimulating activities across the lifespan accounts for a significant amount of variance in depressive thoughts and symptoms in community-dwelling older people. The results indicate that there was a small negative association of lifetime cognitive activity with depressive thoughts and a moderate negative association with depressive symptoms, in that greater experience of an active cognitive lifestyle was associated with fewer depressive thoughts and symptoms. The second aim of the current study was to assess whether there was a cumulative effect of the individual components of an active cognitive lifestyle, namely education, occupational complexity and participation in cognitively stimulating leisure activities at different times across the lifespan, accounting for variance in depressive thoughts and symptoms. The results indicate that the percentage of variance accounted for in both thoughts and symptoms increased with the addition of the elements of the cognitive lifestyle score for each life stage into the model. However, only engagement in cognitive activities in later life was an independently significant predictor for both depressive thoughts and symptoms in the full model. This suggests that the association between current activity participation, a more proximal life experience, and depressive thoughts and symptoms outweighs any association with earlier, more distal, life experiences. The path analysis model specifies the direct pathways between early life experiences and mid-life experiences, and from mid-life experiences to late-life activities. This contribution of early and mid-life experiences to later activity, and the associations found between early and mid-life experiences and depressive thoughts and symptoms in the correlation analysis and in the regressions before later life activities were added, suggest that these early life experiences may contribute to depressive thoughts and symptoms in later life. These inter-relationships also demonstrate the importance of considering these associations in the context of a lifespan perspective. Additionally, it is possible that the current level of participation in activities has a reciprocal relationship with the current level of depressive thoughts and symptoms, whereas it is less probable that the associations with earlier life experiences are bi-directional given that these experiences occurred a long time before the assessment of depressive thoughts and symptoms.

The amount of variance in both depressive thoughts and symptoms explained by the established measure of lifetime cognitive activity was lower than that explained by summing the amount explained by each of the individual elements that it purports to assess. It is probable that this is an artefact of the scoring methods for this measure, which give an equal weighting to experiences in young, mid and later life (Valenzuela and Sachdev 2007); when the experiences were considered individually, later life cognitive activities had the strongest association with depressive thoughts and symptoms. However, for future research that seeks to understand the association of cognitive reserve, which is thought to be accumulated throughout life (Nucci et al. 2011; Richards and Deary 2005; Richards and Sacker 2003; Sánchez Rodríguez et al. 2011; Stern 2009; Tucker and Stern 2011; Whalley et al. 2006), with mood and well-being in later life, the total score on this validated measure of lifetime experiences offers a useful index of the key life experiences thought to increase cognitive reserve.

The results of the current study were similar to those of previous research in that all of the cognitively stimulating life experiences were associated with depressive symptoms in later life (e.g. Adams et al. 2011; Alvarado et al. 2007; Bjelland et al. 2008; Glass et al. 2006; Hong et al. 2009; Jenkins 2011; Ladin 2008; Lorant et al. 2003; Murrell et al. 2003; Narushima et al. 2013; Ross and Mirowsky 2006, 2010). However, several previous studies have noted an independent association of education with depressive symptoms in later life when other life experiences are accounted for (Bjelland et al. 2008; Ladin 2008; Ross and Mirowsky 2006), which was not the case in the current study. Education showed a small negative association with depressive symptoms and was not an independently significant predictor of either depressive thoughts or symptoms when other life experiences were considered. This study is novel in examining, through a lifespan perspective, how cognitive activities throughout life are related to depressive thoughts and symptoms individually and in combination with each other. The results of the current study suggest that there is an indirect positive pathway between cognitive lifestyle experiences in early and mid-life and depressive thoughts and symptoms through current life experiences. While this is the first study to focus on cognitive activity, it is well established that earlier life experiences such as childhood socioeconomic status have an indirect pathway to later life depression through experiences in mid- and later life (e.g. Bjelland et al. 2008; Blane et al. 2012; Murrell et al. 2003; Platts et al. 2015; Ross and Mirowsky 2010). The suggestion that there may be a common pathological process underlying depression and dementia (e.g. Byers and Yaffe 2011; Korczyn and Halperin 2009; Leonard 2007) could help to explain why indicators of cognitive reserve are associated with fewer depressive symptoms and thoughts as well as a reduced risk of cognitive decline and dementia. Further research utilising samples with clinically diagnosed depression and imaging methods to assess structural and functional commonalities is needed to determine whether this is the case.

While the current study suggests that cognitive reserve, as indicated by lifetime cognitive activity, is associated with less experience of depressive thoughts and symptoms in later life as well as better cognitive health, there are some limitations to be considered. The primary limitation of this study is that participants were required to retrospectively recall their levels of activity, which may not give a fully accurate indication of the activities they participated in. Additionally, those who have more depressive thoughts or symptoms may have a more negative view of their past and may fail to accurately identify all the activities they have undertaken. Future research could adopt longitudinal designs making it possible to utilise objective as well as self-report measures. As participants were drawn from the community and were self-selecting, it may not be possible to fully generalise the results to the whole community-dwelling population, as those with lower levels of participation and higher levels of depressive thoughts and symptoms may not have been willing to participate. However, this is a criticism that could be levelled at many studies of community-dwelling older people. The participants in this study had low levels of depressive symptoms and consequently it is not possible to determine whether higher levels of cognitive reserve are associated with less clinical depression. Future studies could help elucidate the results by assessing the associations in those with clinical depression. However, while depression was rare, depressive thoughts were more frequently observed, in some cases at a clinical level, and these are thought to increase the risk of depression (Beck 2002; Evans et al. 2005; Zauszniewski 1997; Zauszniewski and Rong 1999). The negative associations of these negative thoughts with occupational complexity and engagement in cognitive activities across the lifespan provide evidence that there may be a role for cognitive reserve in the thought processes associated with depression. It should also be noted that the associations between the key proxy measures of cognitive reserve and depressive thoughts and symptoms were modest and there may be a number of other factors that play a greater role in the experience of depressive thoughts and symptoms in later life, such as health, bereavement and low socioeconomic status (Alexopoulos 2005; Blazer 2003; Schoevers et al. 2006).

## Conclusion

The associations seen in this sample of community-dwelling older people between greater participation in cognitive activities, especially in later life, and fewer depressive thoughts and symptoms suggest that preventive interventions aimed at increasing participation in cognitively stimulating activity could be beneficial in decreasing the risk of depression in later life. There are benefits of physical activity interventions on depressive symptoms and clinical depression in older people (Blake et al. 2009); similar intervention strategies could be utilised to investigate the effect of increasing cognitive activity on depression. As it has previously been demonstrated that cognitive reserve is also of value in maintaining cognitive health in later life, any increase in the activities associated with building cognitive reserve could have manifold benefits for older people in helping to maintain both cognitive and psychological well-being.
